# Health Monitoring and *Life on the Mississippi*

**Published:** 2004-03-15

**Authors:** Lynne S. Wilcox

Designing health monitoring systems is a complex task. This issue of *Preventing Chronic Disease* includes a report and commentary on measuring the burden of diabetes at the individual level in minority populations (1, 2) and a report on measuring heart disease and stroke indicators at the policy level (3).

To inspire stalwart professionals to design such systems, I turn to an individual recognized for his insightful commentary — Mark Twain, also known as Samuel Clemens. Twain had a keen eye for the idiosyncrasies of human behavior, and his nonfiction works suggest he was adept at amateur qualitative research. Though he was a man of letters rather than a scientist, he clearly appreciated the issues involved in gathering quality information:

There is something fascinating about science. One gets such wholesome returns of conjecture out of such a trifling investment of fact (4).

The balance of conjecture and fact is a source of ongoing tension in public health: collecting data is time-consuming and costly, but operating health programs based on conjecture is risky.

Although *Healthy People 2010* (5) emphasizes the elimination of health disparities, the nation lacks an accurate way of measuring the burden of diabetes in minority populations. Surveillance systems, for example, may treat Spanish-speaking populations as a homogeneous Hispanic group while individuals within the group may have originated from different Spanish-speaking countries (1). Without an understanding of the diversity of these cultures, health programs may lack the context to serve these populations effectively. A report in this issue of *Preventing Chronic Disease*, prepared by an expert panel at the Centers for Disease Control and Prevention (CDC), recommends extending the capacity of existing surveys to obtain better measurements of minority populations instead of developing new surveys, in light of the high costs of taking the latter route (1).

Community-level policy and environmental indicators related to stroke and heart disease prevention present a different problem. Researchers in 2 states, Alabama and South Carolina, examined data sources for 31 pilot indicators and found that, while data sources for most indicators are available in the school setting and are available for indicators of tobacco policies across all settings examined, data sources are least available in the health care and work site settings(3). This report calls for combining current data sources with new surveillance efforts. These efforts are also likely to be costly, but will focus on the policies of states, work sites, and health care organizations, rather than on data from individual respondents.

There are three kinds of lies: lies, damned lies and statistics (6).

The game of "lying with statistics" creates confusion for both public health professionals and the people who use their data. Even when diligent public health professionals strive to explain the nuances of surveillance findings, numbers are often misconstrued by well-meaning policy makers.

The CDC expert panel on using survey data for diabetes surveillance among minority populations points out that most diabetes surveys lack sufficient sample size to provide statistics for the burden of diabetes among smaller minority populations, especially at state or local levels. These populations thus remain hidden and the causes of health disparities remain obscure. The panel recommends enhancing community-level health surveys to gain more details on populations within each community.

The study on policy indicators for heart disease and stroke identifies another challenge in surveillance statistics: indicators may lack the sensitivity or specificity needed to assess a policy's effects on health (3). The study's investigators define an insensitive indicator as one that states the simple presence or absence of a policy without indicating the extent to which the policy addresses an issue. Indicators with poor specificity are ambiguous or lack definition of key terms. The report found that both characteristics were frequent problems among the indicators examined. The authors state that improving sensitivity and specificity of indicators is critical to measuring improvements in population health.

I was gratified to be able to answer promptly, and I did. I said I didn't know (7).

There are times when humility is the best response. The expert panel and policy indicator research group labored long and hard to establish recommendations. Nevertheless, the recommendations are still only conjecture, and until they are implemented and validated, we must fall back on Twain's prompt response as a young riverboat pilot.

When we have no answers, we can contemplate quotes from a master. Indeed, these quotes may carry you through any number of trying circumstances in public health. Twain was a pragmatic man. He would understand if you quote him frequently, and occasionally without proper reference.
